# Eating disinhibition and vagal tone moderate the postprandial response to glycemic load: a randomised controlled trial

**DOI:** 10.1038/srep35740

**Published:** 2016-10-20

**Authors:** Hayley A. Young, Heather Watkins

**Affiliations:** 1 Department of Psychology, Swansea University Swansea, SA2 8PP Wales, UK

## Abstract

Reducing the glycemic load (GL) of the diet may benefit appetite control but its utility is complicated by psychological influences on eating. Disinhibited behaviour, a risk factor for overconsumption, is characterized by reduced prefrontal cortex activity, which in turn modulates vagal tone; a phenomenon associated with glucoregulation. This double blind randomised controlled trial explored for the first time the influence of disinhibited eating and vagal tone (heart rate variability (HRV)) on hunger and the postprandial response to GL. Blood glucose (BG) and hunger were measured 30 and 150 min after consumption of water, glucose or isomaltulose (low glycemic sugar). After consuming glucose, independently of BMI or habitual diet, those with the highest levels of disinhibition had higher BG levels after thirty minutes (B = 0.192, 95% CI LL. 086, UL 0.297), and lower BG after one hundred and fifty minutes (B = −0.240, 95% CI LL −0.348, UL −0.131). BG was related to hunger but only in low disinhibited eaters. Disinhibited eaters were characterised by a reduced HRV which was related to greater BG excursions (B = 0.407, 95% CI LL 0.044, UL 1.134). These findings highlight novel mechanisms by which disinhibited eating leads to obesity and insulin resistance. This trial was registered at clinicaltrials.gov NCT02827318.

The glycemic load (GL) defines meals according to their postprandial glycemic impact^1^; a high GL meal would be expected to produce a hyperglycemic response within the first postprandial hour, followed by a rapid fall in blood glucose (BG) two to four hours later. There is growing recognition that lowering the glycemic load of the diet might reduce a range of cardiovascular risk factors such as raised plasma triglycerides, HbA1c and C reactive protein^2^,^3^ and aid in body weight regulation^4^. A proposed mechanism includes higher satiety and prolonged satiation by virtue of improved postprandial metabolic control^5^, although, whether lower GL meals result in greater weight loss or increased satiety is still a matter of debate^6^,^7^,^8^. One matter complicating the issue is that the desire to consume food may be driven by psychological factors; food reward centres in the brain may override hormonal regulation of food intake^9^. Amongst psychological factors eating disinhibition (the propensity to eat opportunistically in response to emotional and environmental cues) has the largest and most consistent body of empirical data that associates it with weight gain^10^. Indeed a review of seven prospective trials have supported the association between BMI and disinhibition^10^, however, the mechanisms driving eating disinhibition are still unknown. In the present study we consider the possibility that, irrespective of BMI or habitual diet, disinhibited eaters have altered vagal tone and greater glycemic excursions following a high glycemic load drink; effects that compromise subsequent satiation.

The negative health consequences of disinhibited eating are generally considered the result of poor dietary choices^11^, as such the possibility that disinhibition directly influences the postprandial response to a meal has not been considered. Nonetheless, from a neurobiological perspective there is reason to suspect that there might be a direct link: Dietrich, *et al*.^12^ recently reported that a disinhibited eating style correlated negatively with the strength of functional connectivity between the dorsomedial prefrontal (dmPFC) cortex and the amygdala and caudate. It is noteworthy that such regions are also commonly associated with individual differences in heart rate variability (HRV)^13^; an indicator of vagal tone. For instance, HRV has been found to covary with the activity of the medial prefrontal cortex, anterior cingulate cortex, parts of the basal ganglia and the amygdala^14^,^15^ (See ref. 16 for further information on the neural connections between vmPFC and the vagus nerve). Indeed, vagal tone, as measured by HRV, is often considered a biomarker of one’s capacity to maintain inhibitory control^13^,^17^,^18^,^19^. Taken together these findings suggest that disinhibited eaters may, by virtue of altered PFC activity, be characterised by reduced vagal tone. One aim of the present study was to test this hypothesis.

Beyond an index of self-regulatory capacity, the vagus nerve is known to have a wide range of metabolic and anti-inflammatory effects, for example, the vagus nerve is a main component of the cholinergic anti-inflammatory pathway^20^. In addition, low vagal tone, as measured by HRV, has been shown in prospective trials to predate the development of diabetes. Carnethon *et al*.^21^ examined the prospective association between HRV and the development of diabetes in 8,185 middle-aged men and women from the ARIC study. The follow up period lasted eight years during which 1,063 participants developed diabetes. Compared to those in the highest quartile of HRV, those in the lowest quartile had a 1.2-fold greater risk of developing diabetes after adjustment for age, race, gender, education, alcohol use, smoking, heart disease, physical activity and BMI. In addition, a recent study found that declines in HRV statistically mediated the effect of work stress on fasting glucose and HbA1c^22^, supporting a role for psychological factors in modulating vagal tone with consequences for metabolism.

Taken together these finding suggested that disinhibited eaters might, via differences in vagal activity, experience an altered postprandial milieu hindering subsequent appetite control. Therefore, the present study sought to determine the influence of disinhibited eating on the postprandial glycemic response to drinks differing in GL and whether this was related to hunger. Also considered was whether reduced vagal tone in disinhibited eaters might account for differences in glycemia. Accordingly, after fasting overnight young adults consumed glucose, isomaltulose (a low glycemic index (GI) carbohydrate) or water and had their blood glucose and state hunger monitored for the next two and a half hours. Measures of eating disinhibition, food frequency, BMI and resting HRV were obtained. Our results highlight a specific role for disinhibited eating in the management of the postprandial glycemic response; over and above the effect of overeating, such mechanisms may play a role in the link between disinhibited eating and obesity.

## Results

### The effect of disinhibited eating on the postprandial response to GL

**Figure 1:**
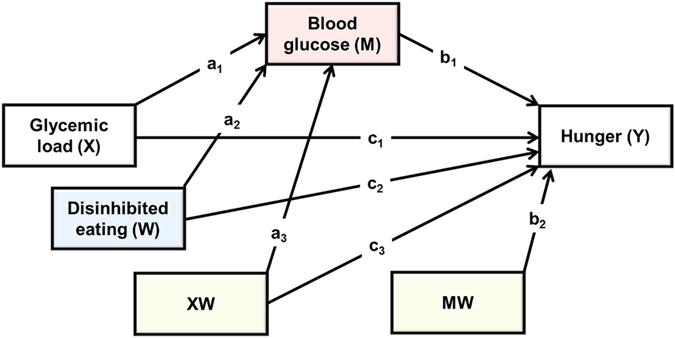
The statistical model which tested the effects of disinhibited eating on the postprandial response to GL and subsequent hunger.

To determine the effect of GL on blood glucose (BG) and hunger two sets of change scores were calculated. BG taken after thirty minutes minus BG at baseline was taken as in index of the raise in BG levels during the early post prandial period. BG after one hundred and fifty minutes minus BG after thirty minutes was taken as an index of the speed of decline from the zenith to the nadir. Parallel scores were calculated for ratings of hunger. A moderated mediation analysis was used to determine the influence of disinhibited eating on the postprandial response to GL, and to establish the consequences for subsequent hunger (Hayes PROCESS model 59; Fig. 1)^23^. Dummy variables were created for type of drink and these were considered in turn as independent variables (X): as such the analysis considered the relative direct and indirect effects of consuming glucose rather than isomaltulose when controlling for water. Hunger was the dependent variable (Y), and blood glucose level was the mediator (M). Disinhibited eating was considered a moderator (W) of both the direct and indirect effects, and BMI was considered as a covariant of both M and Y.

### After thirty minutes

**Figure 2:**
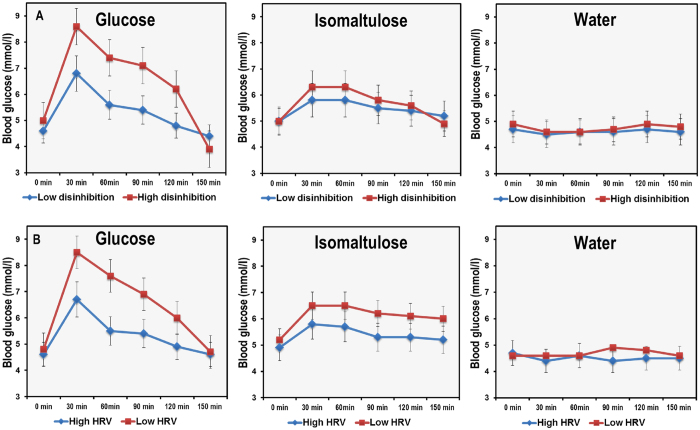
Top: The effect of consuming glucose, isomaltulose or water on postprandial blood glucose in high and low disinhibited eaters. Bottom: The effect of consuming glucose, isomaltulose or water on postprandial blood glucose in those with high and low HR entropy. Data are mean (se) for blood glucose levels during the postprandial period (N = 66). After thirty minutes disinhibited eaters had a greater increase in blood glucose levels if they had consumed glucose (B = 0.192, 95% CI 0.086, 0.297). In addition those high in disinhibited eating had an abrupt decline in blood glucose from thirty to one hundred and fifty minutes after consuming glucose (B = −0.240, 95% CI −0.348, −0.131). Disinhibition did not modulate the postprandial glycemic profile after either water or isomaltulose. Similarly, those with a lower HR entropy had a higher zenith after thirty minutes (B = −14.489, 95% CI LL −26.019, UL −2.959) if they consumed glucose – their blood glucose also fell more quickly (B = 6.180, 95% CI LL 0.842, UL 23.914).

Initially the effects of GL, disinhibition and their interaction on blood glucose were considered (paths a_1_, a_2_ and a_3_; Fig. 1). The model was significant (R^2^  = 0.71, F(5,52) = 26.512, p < 0001) accounting for 71% of the variance in BG. As expected there was a main effect of consuming glucose relative to isomaltulose (B = 1.695, 95% CI LL 1.202, UL 2.187); those who consumed glucose had higher blood glucose. There was also a main effect of disinhibition (B = 0.079, 95% CI LL 0.027, UL 0.130); the greatest blood glucose levels were experienced by those with the highest levels of disinhibition. Interestingly, the interaction between GL and disinhibition was also significant (B = 0.192, 95% CI LL. 086, UL 0.297). The effect was that those with the highest levels of disinhibited eating had the highest blood glucose levels after consuming glucose (Fig. 2). Importantly, this was true even after controlling for the effect of BMI which did not predict blood glucose levels after thirty minutes (B = 0.044, 95% CI LL −0.016, UL 0.105).

Next the effects of GL, blood glucose levels and disinhibition on hunger after thirty minutes were considered (paths c_1_, c_2_, c_3_, b_1_ and b_2_; Fig. 1). The model accounted for 20% of the variance in hunger and was significant (R^2^ = 0.20, F(7,50) = 2.014, p < 05). The main effects of GL (B = −6.292 95% CI LL −16.465, UL 3.881) and BG level (B = 0.319, 95% CI LL −3.676, UL 4.316) did not predict hunger. However, there was a main effect of disinhibition (B = 1.501, 95% CI LL 0.587, UL 2.415); after consuming a drink those highest in disinhibited eating had an increase in hunger whereas those lower had a decrease in hunger. The interactions BG X Disinhibition (B = −0.142, 95% CI LL −0.840, UL 0.556) and GL X Disinhibition (B = 0.244, 95% CI LL −2.296, UL 2.784) were not significant and BMI did not predict hunger (B = 0.412, 95% CI LL −0.585, UL 1.410).

Finally the moderated indirect effect of GL on hunger through blood glucose levels was examined (path a_3_b_1_; Fig. 1). Change in BG predicted the change in hunger (B = 3.338, 95% CI LL 0.598, UL 6.079) and BG mediated the effect of consuming glucose relative to isomaltulose or water on hunger (B = 3.262, 95% CI LL 0.088, UL 5.981) but this did not depend on the level of disinhibition.

#### After one hundred and fifty minutes

The model which considered the effect of GL, disinhibition and their interaction on change in BG was significant (R^2^  = 0 .78, F(5,53) = 39.309, p < 0.0001) accounting for 78% of the variance. Consuming glucose rather than isomaltulose resulted in a sharper decline in BG levels (B = −2.242, 95% CI LL −0.186, UL −1.740) during the late postprandial period, an effect that depended upon the level of disinhibition (B = −0.240, 95% CI LL −0.348, UL −0.131): consuming glucose rather than isomaltulose resulted in a steeper decline in BG levels in highly disinhibited eaters (Fig. 2). BMI did not predict BG levels (B = −0.063, 95% CI LL −0.126, UL −0.001).

When the effects of GL, blood glucose levels and disinhibition on hunger were considered, the model accounted for 32% of the variance in hunger and was significant (R^2^  = 0 .32, F(7,51) = 3.493, p < 0.003). Consuming glucose rather than isomaltulose resulted in greater hunger (B = 28.268, 95% CI LL 10.728, UL 45.807) but the interaction GL X disinhibition was not significant (B = 0.185, 95% CI LL −4.203, UL 4.574). There was no main effect of BG (B = −0.122, 95% CI LL −5.551, UL 5.307), however, the interaction BG X disinhibition reached significance (B = −0.446, 95% CI LL −2.202, UL −0.371); blood glucose only predicted hunger in those low in disinhibition. BMI was not (B = −0.343, 95% CI LL −1.859, UL 1.172) related to hunger.

**Figure 3:**
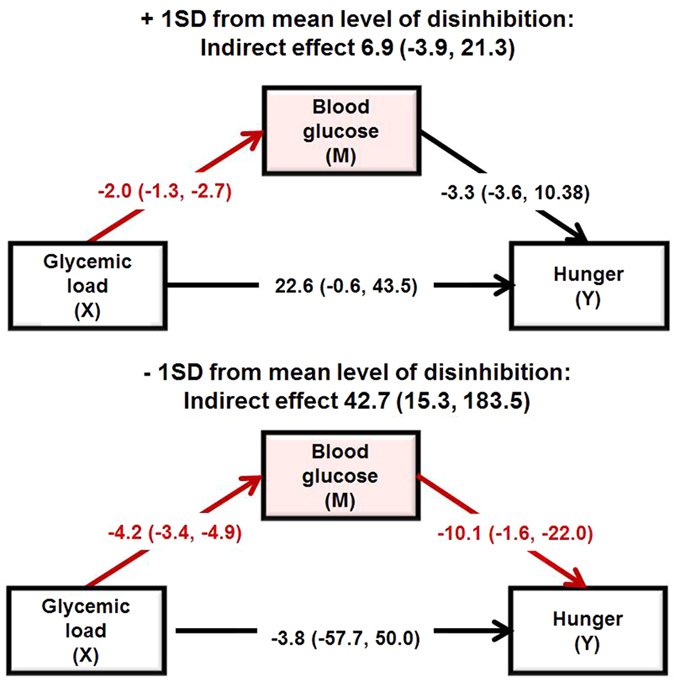
Moderated indirect effects of glycemic load (GL) on hunger through changes in blood glucose depending on level of disinhibited eating during the late postprandial period. Data are B (95%CI) for +/− 1SD away from the mean for disinhibition. (N = 66). The analysis compared glucose to isomaltulose whilst controlling for water. Blood glucose mediated the effect of GL on hunger but only in low disinhibited eaters. Significant paths are highlighted in red.

When the moderated indirect effect of GL on hunger through blood glucose levels was considered, blood glucose mediated the effect of GL on hunger but only in low disinhibited eaters (Fig. 3). Consuming isomaltulose rather than glucose reduced hunger at the end of the morning but only in low disinhibited eaters.

### The effect of vagal tone

**Figure 4:**
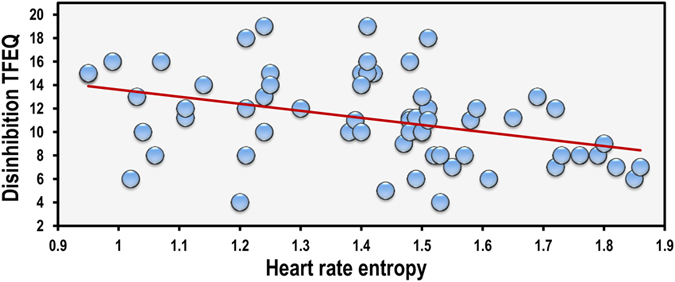
The association between disinhibited eating and heart rate entropy. Data are resting heart rate entropy and disinhibition scores from the three factor eating questionnaire. N = 66. Those high in disinhibited eating had a lower SampEn (B = −6.308, p < 0.004).

It is possible that that postprandial differences in disinhibited eaters results from alterations in vagal tone. Initially baseline scores were examined to determine whether disinhibited eaters had lower heart rate variability while fasted. A hierarchical regression was conducted: step one controlled for BMI and step two included the average RR interval, HFpow and SampEn. Disinhibited eating was the dependant variable. Data are shown in Table 1 and Fig. 4. Those high in disinhibited eating had a lower SampEn (B = −6.308, p < 0.005), that is they had a lower heart rate complexity. In addition, highly disinhibited eaters also had lower vagal activity as indexed by the HFpow (B = 0.022, p < 0.03) and tended to have a higher HR (lower RR interval), although this effect did not reach significance (B = −.015, p < 0.07).

**Figure 5:**
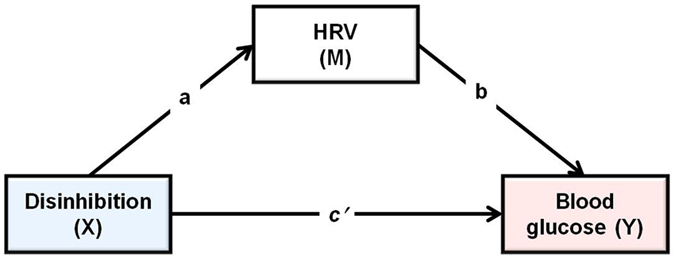
The statistical mediation model which tested the effects of disinhibited eating on heart rate variability (HRV) and blood glucose levels.

To explore the possibility that reduced HRV contributes to the altered glucose metabolism in disinhibited eaters, a multiple mediation analysis was carried out using Hayes PROCESS macro for SPSS model 4^23^. To exclude the confounding influence of GL this analysis was conducted only in those who consumed glucose (n = 21). Disinhibited eating was considered as the independent variable (X), change in BG level was used as the dependent variable (Y), and HRV (RR interval, HFpow and SampEn) were mediators (M) (Fig. 5). BMI was entered as a covariate of both M and Y.

#### After thirty minutes

Overall the model was significant (R^2^  = 0 .86, F(5,12) = 5.198, p < 0.05). Interestingly all three HRV indices independently predicted BG: RR interval (B = −0.018, 95% CI LL −0.035, UL -−0.001), HFpow (B = 0.003, 95% CI LL 0.005, UL 0.055), SampEn (B = −14.489, 95% CI LL −26.019, UL −2.959). Notably, when accounting for HRV, disinhibition was not related to the change in BG (B = −0.217, 95% CI LL −0.602, UL 0.168); importantly this was after controlling for BMI which did not relate to BG (B = −0.099, 95% CI LL −0.338, UL 0.139).

When the indirect effect of disinhibition on change in BG was considered SampEn accounted for the effect, as did the RR interval (B = 0.113, 95% CI LL 0.004, UL 0.564). The indirect effect of HFpow (B = −0.073, 95% CI LL −0.346, UL 0.163) was not significant. The effect was that those highest in disinhibition had a lower HR complexity and a higher HR which in turn predicted a greater glycemic response.

#### After one hundred and fifty minutes

**Figure 6:**
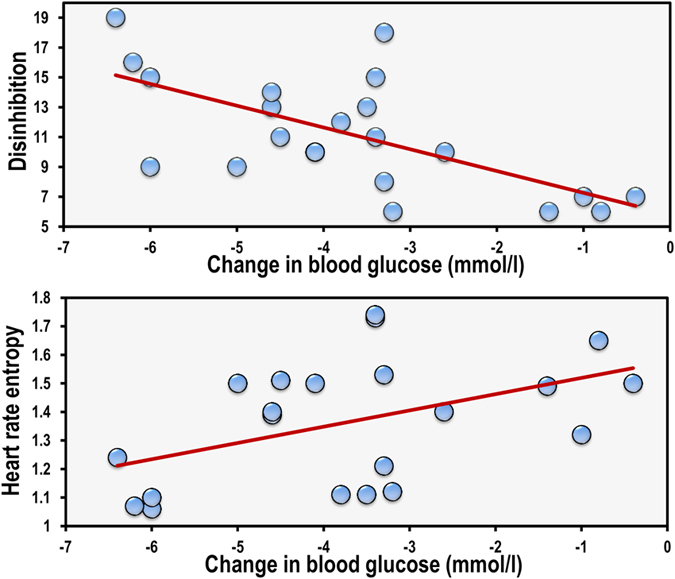
The associations between disinhibited eating change in blood glucose and heart rate complexity. Data are resting heart rate entropy, disinhibition scores from the three factor eating questionnaire and change in blood glucose (after one hundred and fifty minutes minus after thirty minutes) (N = 21). Disinhibited eaters had a lower heart rate complexity (B = −5.80, 95% CI −9.80, −1.81) which in turn was related to a sharper decline in blood glucose levels (B = 6.228, 95% CI 2.431, 10.025).

Similar effects were evident during the late postprandial period; having a steep decline in BG was associated with lower HRV complexity at baseline (B = 6.228, 95% CI LL 2.431, UL 10.025) although neither RR interval (B = 0.005, 95% CI LL −0.007, UL 0.018) nor HFpow B = −0.002, 95% CI LL −0.003, UL 0.001) were related to BG. The only indirect effect to reach significance was that of SampEn which was related to both disinhibition and BG (B = 6.180, 95% CI LL 0.842, UL 23.914); disinhibited eaters had a lower heart rate complexity which was related to having a steeper decline in BG and a lower nadir (Fig. 6).

### The effect of habitual diet

Although the initial analysis controlled for an effect of BMI, disinhibited eaters are known to make less healthy food choices. As such it is possible that the effect of disinhibition on BG levels may be explained by habitual dietary intake. To explore this possibility the above moderated mediation analyses was repeated controlling for habitual diet (see methods section) rather than BMI. Disinhibited eaters did eat a poorer diet (B = −6.135, 95% CI LL −9.777, UL −2.493), however, the overall pattern of results was similar. When controlling for habitual diet disinhibited eaters still had a higher glycemic response to glucose after thirty minutes (GL X Disinhibition: B = 1.792, 95% CI LL 0.625, UL 0.293) and a sharper decline in BG (GL X Disinhibition: B = −0.209, 95% CI LL −0.323, UL −0.095). After 150 minutes consuming a poorer diet habitually was related to BG levels (B = −0.268, 95% CI LL −0.517, UL −0.019) but this effect was independent of the effects of disinhibition – in fact whereas disinhibition was associated with lower BG during the late postprandial period, eating a poorer habitual diet was associated with a higher BG.

To exclude the possibility that disinhibited eaters have a reduced HRV due to a poorer diet the hierarchical regression was repeated controlling for diet. Both SampEn (B = −4.906, 95% CI LL −8.268, UL −1.544) and HFpow (B = 0.002, 95% CI LL 0.001, UL 0.004) remained associated with disinhibition.

Finally, given that disinhibited eaters habitually ate a poorer diet, and that habitual diet was related to BG levels after 150 minutes, the possibility that habitual diet was responsible for the effect of disinhibition on BG was considered. The indirect effect was significant (B = −0.118, 95% CI LL −0.431, UL −0.017), however, the direct effect also remained significant (B = −0.187, 95% CI LL −0.337, UL −0.036) implying partial mediation.

## Discussion

For the first time this study explored the hypothesis that disinhibited eaters have an altered postprandial response to glycemic load (GL). Thirty minutes after consuming a high GL drink those high in disinhibited eating had higher blood glucose (BG) levels, whereas after one hundred and fifty minutes they had the lowest BG levels (Fig. 2). The effect of disinhibition on BG was related to differences in vagal tone; disinhibited eaters had a lower HRV which was associated with having a greater glycemic response during the early post prandial period (PPP) but lower BG during the late PPP (Figs 4 and 6). Importantly, these effects did not depend on BMI or habitual dietary intake; that is disinhibited eaters were characterised by reduced vagal tone and greater glycemic excursions after the influence of BMI and diet had been controlled.

A key finding was that disinhibited eaters were characterised by reduced vagal tone, as measured by HRV (Table 1). This is consistent with a body of evidence that has found HRV to be related to performance on a range of tasks associated with self-regulatory capacity^18^. For example, a recent meta – analysis of 26 studies found that HRV was a significant correlate of self – control^18^. The Neurovisceral Integration Model^16^ posits that trait (i.e., at rest) HRV is a proxy for the ‘inhibitory capacity’ of a central autonomic network (CAN) that regulates behavioural, cognitive, and emotional responses. Brain regions of the CAN comprise those related to executive function and inhibition such as the PFC which exert inhibitory control on subcortical structures and the peripheral nervous system via the vagus nerve^13^. Recent neuroimaging studies have begun to investigate the neural underpinnings of disinhibited eating and find structural and functional changes within the CAN^12^,^24^. Taken together with the present findings it is plausible that disinhibited eaters have reduced vagal tone due to reduced activation of the PFC, although studies in disinhibited eaters combining fMRI and HRV are needed to confirm this hypothesis.

A second important finding was that those high in disinhibited eating experienced greater glucose excursions during the PPP after the high GL (Fig. 2); an effect ameliorated by consuming the low isomaltulose based drink. In line with previous research^21^,^25^,^26^ these changes in BG were negatively related to vagal tone. There are a number of possible explanations for why changes in blood glucose might be attributable to the ameliorated parasympathetic tone in disinhibited eaters. Firstly, the vagus nerve plays a major role in the inflammatory reflex: a neural reflex mechanism in which afferent vagus signalling (activated by cytokines or pathogen-derived products) is associated with efferent vagal output regulating cytokine production and inflammation^20^. For example, a recent study found that vagus nerve-stimulation (VNS) in epilepsy patients inhibits peripheral blood production of TNF, IL-1β, and IL-6^27^. Given that inflammatory processes are thought increase allostatic load^28^ and play a role in the aetiology of insulin resistance^29^, it is possible that chronically reduced vagal tone in disinhibited eaters may predispose towards insulin resistance.

The pattern of changes in BG following the high GL in disinhibited eaters would be consistent with the view that they had mild insulin resistance. Following a glucose load insulin secretion occurs in a biphasic pattern; the first phase, a rapid release that last only a few minutes, is followed by a steady sustained release (second phase)^30^. Loss of first phase glucose stimulated insulin release is found in the early stage of insulin resistance, while the second phase is reduced as diabetes develops^30^,^31^,^32^. The steep rise, followed by a sharp fall in BG (Fig. 2) that was observed in the present study might be attributed to an inadequate early insulin release, although further research measuring plasma insulin is required to confirm this suggestion.

Notably, vagal efferent innervation of the pancreas contributes to early-phase insulin release as well as to optimizing postprandial insulin release^33^. For instance, electrical stimulation of the vagus nerve elicits insulin secretion in different species^34^,^35^. Conversely, atropine that blocks vagal action, significantly reduces basal and stimulated levels of insulin in rats^36^, primates^37^ and humans^38^. In addition, crosstalk between the brain and the liver via the vagus nerve contributes significantly to BG regulation. Vagal activation at the level of the liver inhibits enzymes involved in gluconeogenesis and activates enzymes promoting glycogen synthesis. For instance, atropine or severance of the vagus nerve results in an increase in hepatic glucose production^39^. In return, vagal afferents in the hepatic portal contain glucagon-like peptide-1 receptors (GLP-1r) that convey information about peripheral glucose status back to the brain. Taken together with the present findings it is plausible that disinhibited eaters, by virtue of reduced vagal tone, may be predisposed to inadequate BG regulation following a high GL. However, as we did not directly manipulate vagal tone causality cannot be determined. Recently, Huang *et al*.^40^ found that over a twelve week period transcutaneous auricular vagus nerve stimulation in humans reduced two-hour plasma glucose levels in patients with impaired glucose tolerance. A similar approach might prove fruitful in future studies examining the association between disinhibited eating, vagal activity and BG regulation.

An important consideration in the present study was the contribution of habitual dietary habits. Disinhibited eaters are more likely to choose high-fat and high-salt foods, processed meat, sweet fruits and vegetables, and sweet, carbonated drinks^41^, and to report a higher intake of sweet foods, ice cream, butter and coffee^42^. This suggests that a less healthy food choice could contribute to the altered vagal tone and glucose metabolism that was observed in disinhibited eaters. Indeed it is possible that vagal afferent signalling is altered in response to a high fat diet, even before the onset of obesity^43^,^44^. In the present study consuming a less healthy diet was associated with poorer BG regulation and reduced vagal tone, however, it did not fully account for their association with disinhibition. This suggests that disinhibited eaters have a pre-existing disposition towards impaired vagal tone and glucose intolerance; an effect that may then be exacerbated by a poor diet.

Finally, a low GL did not reduce hunger during the late postprandial period in disinhibited eaters but did in those low in disinhibition (Fig. 3); an effect mediated by the maintenance of a higher BG level in these subjects. From a homeostatic viewpoint, maintaining a stable postprandial BG level should reduce hunger and meal frequency, and thus improve weight control^6^, however this homeostatic process is often negated by the hedonic desire for food reward^9^. The interaction between these two systems is appreciated by research examining the effects of homeostatic hormones, such as insulin and leptin, on brain regions mediating the rewarding nature of food^45^. Vagal signalling plays an important role in appetite and satiety leading to the suggestion that vagal nerve stimulation/blockade might reduce food intake and weight gain. Preclinical research has found that VNS reduces food intake and/or body weight in rats and other animals^46^ and is associated with weight loss in human epileptic patients^47^, although these effects might be diminished in those who are overweight^48^. Recently, the FDA approved a technique of vagal blocking (VBLOCK) as a weight-loss treatment device in obesity. However, clinical trials have yielded contradictory results^49^,^50^,^51^,^52^. In the present study we found that disinhibition was not only associated with decreased vagal tone but also an increase, rather than a decrease, in hunger during the early PPP. Based on this information we speculate that disinhibited eaters might have reduced interoceptive sensitivity leading them to rely on exteroceptive signals in order to regulate their eating behavior.

The limitations of the present study should be considered. Firstly, as a range of factors such as heart disease and medication might influence HRV, a young healthy sample was chosen for the present study: this approach, however, does limit the generalizability of the results. Secondly, although posteriori mathematical modelling will test if the data structure is compatible with causation, it is not a proof of causation. As such future research should directly manipulate vagal tone and monitor effects.

In conclusion, we report that following a high GL disinhibited eaters have a greater glycemic response than their less inhibited counterparts. This response remained even after controlling for BMI and habitual diet; therefore, it can be viewed as an enduring individual difference that predisposes disinhibited eaters to an unfavourable postprandial environment and a range of negative health consequences. Disinhibited eaters were characterised by reduced vagal tone (HRV); an effect associated with larger glycemic excursions in these subjects. Disinhibition also moderated the effect of glycemia on subsequent hunger; that is they appear to be less sensitive to interoceptive signals. The moderating influence of disinhibition might shed light on the debate surrounding the efficacy of low GL diets for increasing satiety and reducing obesity^6^,^7^,^8^. Future research examining the effects of GL should seek to understand its interaction with psychological factors.

## Methods

### Participants

Sixty six females between 18 and 29 years of age were recruited for this study and took part between the months of September and December 2015. Participants were excluded if they had a cardiovascular or metabolic disorder, gastrointestinal problems, were pregnant, had a current diagnosis of a mood or eating disorder, and/or were taking medications or herbal supplements to manage body weight or control appetite. BMI ranged from 17.15 to 31.24 kg/m^2^. Participants were instructed to refrain from drinking alcohol and taking part in any physical activity within twenty four hours of the study and abstain from consuming any food and drink for at least twelve hours before attending the laboratory. As this was the first study to consider the metabolic and appetite response to GL in disinhibited eaters, power analysis was not feasible. However the sample size was based on similar work that has been conducted in restrained eaters^53^.

### Procedure

Upon entry into the laboratory, after providing their informed consent, the participants completed the disinhibition scale of the Three Factor Eating Questionnaire^54^, a food frequency questionnaire (FFQ) and reported their level of hunger. Participants then had their height, weight and fasting BG measured before baseline R-R interval measurements were recorded while they rested quietly for five minutes. This study employed a parallel designed study as such the participants were then randomly allocated to receive water (n = 22), isomaltulose (n = 23) or glucose (n = 21). The random sequence was computer generated by HY who produced the solutions in sequentially numbered tumblers. Participants were allocated by HW in the order they were recruited. The subjects and HW who met the subjects was blind as to the nature of the meals consumed. At baseline the groups were well matched for ratings of hunger, heart rate variability, fasting blood glucose, disinhibition, habitual diet and BMI (Table 2). Participants were given five minutes to consume the entire beverage following which they relaxed (either reading of watching TV) for thirty minutes before they again rated their hunger and a BG measurement was taken. Over the next one hundred and twenty minutes BG measurements were taken every thirty minutes while participants relaxed. After a total of one hundred and fifty minutes a final BG measurement was taken and hunger reported. The procedure was approved by Swansea University ethics committee (08.25.2015.2) and carried out in accordance with the principles laid down by the declaration of Helsinki 2013. All participants completed the study. This trial was registered at clinicaltrials.gov NCT02827318 on 29/06/16.

### Test drinks

Each drink was 500 ml provided in a clear plastic tumbler. The test drinks contained either 75 g of glucose or 75 g of isomaltulose dissolved in water. The sugar free beverage was sweetened with sucralose to produce a similar sweetness to the other drinks. All the drinks contained 10 ml of lemon juice to increase palatability. The glucose and isomaltulose drinks were designed to be identical in terms of macro-nutrients and appearance but produce a different GL^55^. The glucose, isomaltulose and water drinks provided the following GLs respectively: 75, 24 and 0.

### Hunger

Participants were asked to respond to the question “how hungry are you feeling right now” on a single 100 mm visual analogue scale anchored by “Not at all” and “Extremely”.

### Disinhibition

The tendency towards disinhibited eating was measured using the 16 item disinhibition subscale of the three factor eating questionnaire^54^. This scale measures loss of cognitive control of eating using true-false items (eg “Sometimes when I start eating, I just can’t seem to stop”). In the present sample Cronbach’s alpha for the 16 items was 0.759.

### Habitual diet

The European Prospective Investigation into Cancer and Nutrition Norfolk Food Frequency Questionnaire (EPIC-Norfolk FFQ) (Mulligan *et al*. 2014) was used to collect dietary data. During data collection a common unit or portion size for each food was specified and subjects were asked to indicate on a 9 point scale ranging from ‘never’ to ‘6+ per day’, how often they tend to consume specific foods. Bingham, *et al*.^56^ previously validated this tool by comparing it to a 16-day weighed food record. It has also been validated against nutrient biomarkers^57^. FETA software was then used to further process the data. FETA uses UK based food composition databases to produce nutrient data as well as basic food groups^58^. Importantly this gives rise to food groups that are captured cleanly, for example, fruit juice fraction of a juice drink – which may be only 10% of the total product – counts toward total fruit, but the rest of the beverage counts toward added sugars. Likewise, the skim milk fraction of whole milk counts toward the dairy constituent, but the butterfat in whole milk counts toward calories from solid fat. From these food groups a modified version of the alternate healthy eating index (AHEI) score^59^ was created by taking the sum of 7 component scores [1: fruit; 2: vegetable; 3: ratio of white meat (seafood and poultry) to red meat; 4: ratio of polyunsaturated fatty acids (PUFA) to saturated fatty acids9 SFA); 5: total fiber; 6: nuts and seeds; and 7: multivitamin use]. The score ranged between 2.5 and 67.5 with higher values corresponding to a healthier diet – a healthy diet consisting of one that is high in fruit, vegetables, white meat and fish, PUFA, fibre and nuts and seeds and low in SFA and red meat). This approach was chosen to maintain consistency with other large UK based cohort studies that have examined the influence of dietary patterns on mental and physical health^60^ and previous studies by the current authors.

### Body mass index

Body mass was measured using an electronic scale (Kern KMS-TM, Kenr and Sohn GmbH, Germany) that, to avoid problems associated with movement, took 50 assessments over a 5 second period and produced an average value. Height was measured using a portable stadiometer.

### Blood glucose

Blood glucose was monitored from finger pricks using an ExacTech sensor (Medisense Britain Limited) that using an enzymic method, coupled with microelectronic measurement, which has been shown to be accurate^61^.

### Heart rate variability

As an index of vagal tone heart rate variability was calculated from a R-R interval time series. Participants were fitted with a RS800 Polar heart rate monitor electrode transmitter belt (T61) using conductive gel as recommended by the manufacturer. Interbeat interval measurements were collected using a Polar RS800 HR monitor set to R-R interval mode (Polar Electro, Kempele, Finland) at a sampling rate of 1000 Hz. This instrument has been previously validated for the accurate measurement of R-R intervals and for analysing Heart Rate Variability (HRV)^62^. Participants were seated comfortably and asked to relax for five minutes while the HR time series was recorded.

## Statistical Analysis

### Heart rate variability

R-R interval data were analysed using Kubios HRV Analysis Software 2.0 (The Biomedical Signal and Medical Imaging Analysis Group, Department of Applied Physics, University of Kuopio, Finland)^63^. Data were visually inspected for artefacts caused by ectopic beats, poor conductivity etc. A very low correction threshold was chosen for artefact correction (0.45 from local average) so not to distort natural variability. Less than 1% of beats were identified as artefacts.

Spectral analysis was conducted to transform the time series into the frequency domain. The R-R interval series was converted to equidistantly sampled series by cubic spline interpolation at a rate of 4 Hz. Welsh’s periodogram, which divides the R-R series into overlapping windows, was used to decrease the leakage effect, and the spectrum estimate was obtained by averaging the Fast Fourier Transform (FFT) spectra of these windowed segments. Average spectral power was estimated within the high frequency (HFpow) (0.15–0.4) band, which represents vagal activity. As it has previously been reported that nonlinear complexity indices capture additional information^64^ sample entropy (SampEn) was also calculated. Entropy refers to system randomness, regularity and predictability and allows systems to be quantified by the amount of information within the signal. SampEn has been defined as the negative natural logarithm for conditional properties that a series of data points a certain distance apart, m, would repeat itself at m + 1 where self-matches are not included in calculating the probability. A lower value of SampEn also indicates more regularity in the time series. The computation of sample entropy depends on two parameters; the embedding dimension m and the tolerance r. In the present study these were set as m = 2 and r = 0.2 SDNN. See Young and Benton^64^ for formulae for calculating sample entropy and for a graphical representation. The average R-R interval was calculated as a measure of basic heart rate.

### Moderated mediation

To determine the influence of disinhibited eating on the postprandial response to GL and to establish the consequences for subsequent hunger, moderated mediation analysis was conducted using Hayes PROCESS macro for SPSS model 59^23^. This macro uses bootstrapped sampling to estimate the indirect mediation effect. In the present analysis 5000 bootstrapped samples were drawn with replacement from the dataset to estimate a sampling distribution for the indirect mediation pathway. The total effect of X on Y (denoted by c in Fig. 1), can be expressed as the sum of the direct effect (denoted by c′) and indirect effect, which is the product of the a and b paths (denoted by ab), such that c = c′ + ab. Indirect effects (B) and 95% confidence intervals are reported.

### Hierarchical regression

A hierarchical regression was conducted to determine the association between HRV indices and disinhibited eating after controlling for BMI: step one controlled for BMI and step two included the average RR interval, HFpow and SampEn. Disinhibited eating was the dependant variable.

### Detection of outliers

To detect possible outliers Cook’s Distance^65^ was calculated. The Cook’s Distance reflects the extent to which model residuals would change if a particular subject’s data (in multivariate space) were excluded from the estimated regression coefficient. Larger Cook’s Distance values indicate more influential subjects. The threshold for determining influential observations was set as 4/N in line with previous recommendations^66^. When certain cases had a Cook’s Distance that exceeded this threshold those cases were excluded and the data re-analysed. This did not affect the outcome of any analysis and as such no cases were excluded.

## Additional Information

**How to cite this article**: Young, H. A. and Watkins, H. Eating disinhibition and vagal tone moderate the postprandial response to glycemic load: a randomised controlled trial. *Sci. Rep.* **6**, 35740; doi: 10.1038/srep35740 (2016).

## Figures and Tables

**Table 1:** Results from the multiple regression analysis that assessed the associations between resting HRV (while fasted) and disinhibited eating.

DV	IV	B	R^2^	F	*p*
Disinhibition	**MODEL 1**		0.08	0.375	0.543
	BMI	0.134			0.377
	**MODEL 2**		0.53	4.801	**0.002**
	BMI	0.060			0.693
	RR interval	−0.015			**0.075**
	HFpow	0.022			**0.032**
	SampEn	−6.308			**0.004**
	**Change statistics**	**R^2^ = 0.28, F (3,49) = 6.237 p < 0.001**			

Those high in disinhibited eating had a lower heart rate complexity (SampEn) (p < 0.004) and lower vagal activity (HFpow) (p < 0.03).

**Table 2:** Baseline and individual difference scores for participants who received water, isomaltulose or glucose.

	Glucose	Isomaltulose	Water
Baseline hunger	68.38(7.48)	60.16(6.35)	57.07(7.21)
Baseline BG	4.79(0.11)	4.11(0.10)	4.60(0.11)
Baseline RR	788.85(20.48)	757.28(17.40)	791.67(19.73)
Baseline HFpow	607.75(127.34)	363.24(108.22)	586.00(122.71)
Baseline SampEn	1.38(0.07)	1.38(0.06)	1.43(0.07)
Disinhibition	11.81(0.99)	10.00(0.94)	11.11(0.94)
BMI	22.32(0.70)	21.81(0.66)	23.08(0.66)
Habitual diet	50.37(5.72)	32.27(5.39)	39.16(5.39)
Hunger 30 min	63.06(5.93)	61.00(5.59)	65.75(5.59)
BG 30 min	7.75(0.27)*	6.06(0.25)*	4.55(0.25)*
Hunger 150 min	100.31(5.88)*	73.22(5.55)*	88.50(5.55)*
BG 150 min	4.21(0.12)*	5.17(1.11)*	4.76(0.11)*

Scores for hunger, blood glucose and heart rate variability after thirty, and one hundred and fifty minutes are also provided. Data are mean (se). BG – Blood glucose, RR – RR interval, HFpow – High frequency power, SampEn – Sample Entropy, BMI – Body mass index. *p < 0.001.

## References

[b1] Jenkins D. . Glycemic index of foods: a physiological basis for carbohydrate exchange. The American journal of clinical nutrition 34, 362–366 (1981). 6259925 10.1093/ajcn/34.3.362

[b2] Schwingshackl L. & Hoffmann G. Long-term effects of low glycemic index/load vs. high glycemic index/load diets on parameters of obesity and obesity-associated risks: a systematic review and meta-analysis. Nutrition, Metabolism and Cardiovascular Diseases 23, 699–706 (2013). 10.1016/j.numecd.2013.04.008 23786819

[b3] Emadian A., Andrews R. C., England C. Y., Wallace V. & Thompson J. L. The effect of macronutrients on glycaemic control: a systematic review of dietary randomised controlled trials in overweight and obese adults with type 2 diabetes in which there was no difference in weight loss between treatment groups. British Journal of Nutrition 114, 1656–1666 (2015). 26411958 10.1017/S0007114515003475 PMC4657029

[b4] Du H. . Dietary glycaemic index, glycaemic load and subsequent changes of weight and waist circumference in European men and women. International journal of obesity 33, 1280–1288 (2009). 19704411 10.1038/ijo.2009.163

[b5] Flint A. . Glycemic and insulinemic responses as determinants of appetite in humans. The American journal of clinical nutrition 84, 1365–1373 (2006). 17158418 10.1093/ajcn/84.6.1365

[b6] Ludwig D. S. The glycemic index: physiological mechanisms relating to obesity, diabetes, and cardiovascular disease. Jama 287, 2414–2423 (2002). 11988062 10.1001/jama.287.18.2414

[b7] Bornet F. R., Jardy-Gennetier A.-E., Jacquet N. & Stowell J. Glycaemic response to foods: impact on satiety and long-term weight regulation. Appetite 49, 535–553 (2007). 17610996 10.1016/j.appet.2007.04.006

[b8] Akhavan T. & Anderson G. H. Effects of glucose-to-fructose ratios in solutions on subjective satiety, food intake, and satiety hormones in young men. The American journal of clinical nutrition 86, 1354–1363 (2007). 17991646 10.1093/ajcn/86.5.1354

[b9] Morton G. J., Meek T. H. & Schwartz M. W. Neurobiology of food intake in health and disease. Nature reviews. Neuroscience 15, 367 (2014). 24840801 10.1038/nrn3745 PMC4076116

[b10] French S. A., Epstein L. H., Jeffery R. W., Blundell J. E. & Wardle J. Eating behavior dimensions. Associations with energy intake and body weight. A review. Appetite 59, 541–549 (2012). 22796186 10.1016/j.appet.2012.07.001 PMC3454469

[b11] Bryant E. J., King N. & Blundell J. E. Disinhibition: its effects on appetite and weight regulation. Obesity Reviews 9, 409–419 (2008). 18179615 10.1111/j.1467-789X.2007.00426.x

[b12] Dietrich A., Hollmann M., Mathar D., Villringer A. & Horstmann A. Brain regulation of food craving: relationships with weight status and eating behavior. International Journal of Obesity 40, 982–989 (2016). 26883294 10.1038/ijo.2016.28

[b13] Thayer J. F., Åhs F., Fredrikson M., Sollers J. J. & Wager T. D. A meta-analysis of heart rate variability and neuroimaging studies: implications for heart rate variability as a marker of stress and health. Neuroscience & Biobehavioral Reviews 36, 747–756 (2012). 22178086 10.1016/j.neubiorev.2011.11.009

[b14] Chang C. . Association between heart rate variability and fluctuations in resting-state functional connectivity. Neuroimage 68, 93–104 (2013). 23246859 10.1016/j.neuroimage.2012.11.038 PMC3746190

[b15] Jennings J. R., Sheu L. K., Kuan D. C. H., Manuck S. B. & Gianaros P. J. Resting state connectivity of the medial prefrontal cortex covaries with individual differences in high‐frequency heart rate variability. Psychophysiology (2015). 10.1111/psyp.12586 PMC4800828 26995634

[b16] Thayer J. F. & Lane R. D. Claude Bernard and the heart–brain connection: Further elaboration of a model of neurovisceral integration. Neuroscience & Biobehavioral Reviews 33, 81–88 (2009). 18771686 10.1016/j.neubiorev.2008.08.004

[b17] Williams D. P. . Resting heart rate variability predicts self-reported difficulties in emotion regulation: a focus on different facets of emotion regulation. Frontiers in psychology 6 (2015). 10.3389/fpsyg.2015.00261 PMC4354240 25806017

[b18] Zahn D. . Heart rate variability and self-control—A meta-analysis. Biological psychology 115, 9–26 (2016). 26747415 10.1016/j.biopsycho.2015.12.007

[b19] Appelhans B. M. & Luecken L. J. Heart rate variability as an index of regulated emotional responding. Review of general psychology 10, 229 (2006).

[b20] Pavlov V. A., Wang H., Czura C. J., Friedman S. G. & Tracey K. J. The cholinergic anti-inflammatory pathway: a missing link in neuroimmunomodulation. Molecular Medicine-Cambridge Ma Then New York 9, 125–134 (2003). PMC1430829 14571320

[b21] Carnethon M. R., Golden S. H., Folsom A. R., Haskell W. & Liao D. Prospective Investigation of Autonomic Nervous System Function and the Development of Type 2 Diabetes The Atherosclerosis Risk In Communities Study, 1987–1998. Circulation 107, 2190–2195 (2003). 12695289 10.1161/01.CIR.0000066324.74807.95

[b22] Jarczok M. N. . The Association of Work Stress and Glycemic Status Is Partially Mediated by Autonomic Nervous System Function: Cross-Sectional Results from the Mannheim Industrial Cohort Study (MICS). Plos One 11, e0160743 (2016). 27532642 10.1371/journal.pone.0160743 PMC4988666

[b23] Hayes A. F. Introduction to mediation, moderation, and conditional process analysis: A regression-based approach. (Guilford Press, 2013).

[b24] Maayan L., Hoogendoorn C., Sweat V. & Convit A. Disinhibited eating in obese adolescents is associated with orbitofrontal volume reductions and executive dysfunction. Obesity 19, 1382–1387 (2011). 21350433 10.1038/oby.2011.15 PMC3124611

[b25] Jarczok M. N., Li J., Mauss D., Fischer J. E. & Thayer J. F. Heart rate variability is associated with glycemic status after controlling for components of the metabolic syndrome. International journal of cardiology 167, 855–861 (2013). 22386703 10.1016/j.ijcard.2012.02.002

[b26] Jarczok M. N., Koenig J., Schuster A. K., Thayer J. F. & Fischer J. E. Nighttime heart rate variability, overnight urinary norepinephrine, and glycemic status in apparently healthy human adults. Int J Cardiol 168, 3025–3026 (2013). 23651814 10.1016/j.ijcard.2013.04.147

[b27] Koopman F. A. . Vagus nerve stimulation inhibits cytokine production and attenuates disease severity in rheumatoid arthritis. Proceedings of the National Academy of Sciences 113, 8284–8289 (2016). 10.1073/pnas.1605635113 PMC4961187 27382171

[b28] Shiels P. G., Stenvinkel P., Kooman J. P. & McGuinness D. Circulating markers of ageing and allostatic load: A slow train coming. Practical Laboratory Medicine (2016). 10.1016/j.plabm.2016.04.002 PMC5574864 28856219

[b29] Herder C. . Biomarkers of subclinical inflammation and increases in glycaemia, insulin resistance and beta-cell function in non-diabetic individuals: the Whitehall II study. European Journal of Endocrinology EJE-16–0528 (2016). 10.1530/EJE-16-0528 27491375

[b30] Seino S., Shibasaki T. & Minami K. Dynamics of insulin secretion and the clinical implications for obesity and diabetes. The Journal of clinical investigation 121, 2118–2125 (2011). 21633180 10.1172/JCI45680 PMC3104758

[b31] Polonsky K. Dynamics of insulin secretion in obesity and diabetes. International Journal of Obesity 24, S29–S31 (2000). 10997604 10.1038/sj.ijo.0801273

[b32] Triplitt C. L. Examining the mechanisms of glucose regulation. The American journal of managed care 18, S4–10 (2012). 22559855

[b33] Teff K. L. Visceral nerves: vagal and sympathetic innervation. Journal of Parenteral and Enteral Nutrition 32, 569–571 (2008). 18753395 10.1177/0148607108321705

[b34] Daniel P. & Henderson J. The effect of vagal stimulation on plasma insulin and glucose levels in the baboon. The Journal of physiology 192, 317 (1967). 4963568 10.1113/jphysiol.1967.sp008302 PMC1365559

[b35] Frohman L. A., Ezdinli E. Z. & Javid R. Effect of vagotomy and vagal stimulation on insulin secretion. Diabetes 16, 443–448 (1967). 5339250 10.2337/diab.16.7.443

[b36] Stubbs M. & York D. Central glucocorticoid regulation of parasympathetic drive to pancreatic B-cells in the obese fa/fa rat. International journal of obesity 15, 547–553 (1991). 1938098

[b37] Daniel P. & Henderson J. The effect of atropine on insulin release caused by intravenous glucose in the rhesus monkey. Acta endocrinologica 78, 736–745 (1975). 808062 10.1530/acta.0.0780736

[b38] Harju E. & Nordback I. Effect of atropine on insulin secretion in healthy subjects. Journal of international medical research 15, 167–169 (1987). 3301456 10.1177/030006058701500307

[b39] Yi C.-X., La Fleur S. E., Fliers E. & Kalsbeek A. The role of the autonomic nervous liver innervation in the control of energy metabolism. Biochimica et Biophysica Acta (BBA)-Molecular Basis of Disease 1802, 416–431 (2010). 20060897 10.1016/j.bbadis.2010.01.006

[b40] Huang F. . Effect of transcutaneous auricular vagus nerve stimulation on impaired glucose tolerance: a pilot randomized study. BMC complementary and alternative medicine 14, 1 (2014). 24968966 10.1186/1472-6882-14-203 PMC4227038

[b41] Contento I. R., Zybert P. & Williams S. S. Relationship of cognitive restraint of eating and disinhibition to the quality of food choices of Latina women and their young children. Preventive medicine 40, 326–336 (2005). 15533547 10.1016/j.ypmed.2004.06.008

[b42] Lähteenmäki L. & Tuorila H. Three-factor eating questionnaire and the use and liking of sweet and fat among dieters. Physiology & behavior 57, 81–88 (1995). 7878129 10.1016/0031-9384(94)00210-v

[b43] Troy A. E., Simmonds S. S., Stocker S. D. & Browning K. N. High fat diet attenuates glucose‐dependent facilitation of 5‐HT3‐mediated responses in rat gastric vagal afferents. The Journal of physiology 594, 99–114 (2016). 26456775 10.1113/JP271558 PMC4704508

[b44] Page A. J. Vagal afferent dysfunction in obesity: cause or effect. The Journal of physiology 594, 5–6 (2016). 26724479 10.1113/JP271669 PMC4704507

[b45] Davis J. F., Choi D. L. & Benoit S. C. Insulin, leptin and reward. Trends in Endocrinology & Metabolism 21, 68–74 (2010). 19818643 10.1016/j.tem.2009.08.004 PMC2822063

[b46] Sobocki J., Krolczyk G., Herman R., Matyja A. & Thor P. Influence of vagal nerve stimulation on food intake and body weight-results of experimental studies. Journal of physiology and pharmacology 56, 27 (2005). 16340036

[b47] Burneo J., Faught E., Knowlton R., Morawetz R. & Kuzniecky R. Weight loss associated with vagus nerve stimulation. Neurology 59, 463–464 (2002). 12177391 10.1212/wnl.59.3.463

[b48] Bodenlos J. S. . Vagus Nerve Stimulation and Food Intake Effect of Body Mass Index. Journal of diabetes science and technology 8, 590–595 (2014). 24876624 10.1177/1932296814525188 PMC4455432

[b49] Camilleri M. . Intra-abdominal vagal blocking (VBLOC therapy): clinical results with a new implantable medical device. Surgery 143, 723–731 (2008). 18549888 10.1016/j.surg.2008.03.015

[b50] Shikora S. A. . Intermittent vagal nerve block for improvements in obesity, cardiovascular risk factors, and glycemic control in patients with type 2 diabetes mellitus: 2-year results of the VBLOC DM2 study. Obesity surgery 26, 1021–1028 (2016). 26471783 10.1007/s11695-015-1914-1

[b51] Ikramuddin S. . Effect of reversible intermittent intra-abdominal vagal nerve blockade on morbid obesity: the ReCharge randomized clinical trial. Jama 312, 915–922 (2014). 25182100 10.1001/jama.2014.10540

[b52] Sarr M. G. . The EMPOWER study: randomized, prospective, double-blind, multicenter trial of vagal blockade to induce weight loss in morbid obesity. Obesity surgery 22, 1771–1782 (2012). 22956251 10.1007/s11695-012-0751-8

[b53] Burton-Freeman B. & Keim N. L. Glycemic index, cholecystokinin, satiety and disinhibition: is there an unappreciated paradox for overweight women&quest. International Journal of Obesity 32, 1647–1654 (2008). 18825157 10.1038/ijo.2008.159

[b54] Stunkard A. J. & Messick S. The three-factor eating questionnaire to measure dietary restraint, disinhibition and hunger. Journal of psychosomatic research 29, 71–83 (1985). 3981480 10.1016/0022-3999(85)90010-8

[b55] Young H. & Benton D. The glycemic load of meals, cognition and mood in middle and older aged adults with differences in glucose tolerance: A randomized trial. e-SPEN Journal 9, e147–e154 (2014).

[b56] Bingham S. . Comparison of dietary assessment methods in nutritional epidemiology: weighed records v. 24 h recalls, food-frequency questionnaires and estimated-diet records. British Journal of Nutrition 72, 619–643 (1994). 7986792 10.1079/bjn19940064

[b57] Bingham S. A. . Nutritional methods in the European prospective investigation of cancer in Norfolk. Public health nutrition 4, 847–858 (2001). 11415493 10.1079/phn2000102

[b58] Mulligan A. A. . A new tool for converting food frequency questionnaire data into nutrient and food group values: FETA research methods and availability. BMJ open 4, e004503 (2014). 10.1136/bmjopen-2013-004503 PMC3975761 24674997

[b59] McCullough M. L. . Diet quality and major chronic disease risk in men and women: moving toward improved dietary guidance. The American journal of clinical nutrition 76, 1261–1271 (2002). 12450892 10.1093/ajcn/76.6.1261

[b60] Akbaraly T. N., Sabia S., Shipley M. J., Batty G. D. & Kivimaki M. Adherence to healthy dietary guidelines and future depressive symptoms: evidence for sex differentials in the Whitehall II study. The American journal of clinical nutrition 97, 419–427 (2013). 23283506 10.3945/ajcn.112.041582 PMC3545684

[b61] Matthews D. . Pen-sized digital 30-second blood glucose meter. The Lancet 329, 778–779 (1987). 10.1016/s0140-6736(87)92802-9 2882186

[b62] Nunan D. . Validity and reliability of short-term heart-rate variability from the Polar S810. Medicine+ Science in Sports+ Exercise 41, 243 (2009). 19092682 10.1249/MSS.0b013e318184a4b1

[b63] Tarvainen M. P., Niskanen J.-P., Lipponen J. A., Ranta-Aho P. O. & Karjalainen P. A. Kubios HRV–heart rate variability analysis software. Computer methods and programs in biomedicine 113, 210–220 (2014). 24054542 10.1016/j.cmpb.2013.07.024

[b64] Young H. & Benton D. We should be using nonlinear indices when relating heart-rate dynamics to cognition and mood. Scientific reports 5 (2015). 10.1038/srep16619 PMC4643265 26565560

[b65] Cook R. D. Detection of influential observation in linear regression. Technometrics 19, 15–18 (1977).

[b66] Bollen K. A. & Jackman R. W. Regression diagnostics an expository treatment of outliers and influential cases. Sociological Methods & Research 13, 510–542 (1985).

